# Interplay between Exciton and Free Carriers in Organolead Perovskite Films

**DOI:** 10.1038/s41598-017-15097-y

**Published:** 2017-11-07

**Authors:** Wei Wang, Yu Li, Xiangyuan Wang, Yang Liu, Yanping Lv, Shufeng Wang, Kai Wang, Yantao Shi, Lixin Xiao, Zhijian Chen, Qihuang Gong

**Affiliations:** 1 State Key Laboratory for Artificial Microstructure and Mesoscopic Physics, Department of Physics, Peking University, Beijing, 100871 China; 2 State Key Laboratory of Fine Chemicals, School of Chemistry, Dalian University of Technology, Dalian, Liaoning 116024 China; 3 Collaborative Innovation Center of Extreme Optics, Shanxi University, Taiyuan, Shanxi 030006 China; 4 Department of Physics and Astronomy and Collaborative Innovation Center of IFSA (CICIFSA), Shanghai Jiao Tong University, Shanghai, 200240 China

## Abstract

For highly interested organolead perovskite based solar cells, the exciton and free carriers are the photoproducts in the working layers. In this study, we revealed their two forms of relations depending on heat-annealing condition. In non-annealed films and single crystal, they are in density-dependent dynamical balance (co-existing). For the sufficiently heat-annealed films, they present a significant emissive exciton-carrier collision (ECC). The two relations indicate the emergence of a subgrain morphology within the tetragonal phase of crystal grain, induced by heat annealing process. Such subgrain structure could be assigned to a ferroelastic twinning structure recently found inside the crystal grain of the films. Since the heat annealing is a general procedure in preparing perovskite working layers, we propose that the ECC and subgrain morphology widely exist in real devices. We suggest that the subgrain structure provides another level of morphological basis for in depth understanding high performance of organolead perovskite working layers.

## Introduction

The organolead perovskites are currently the most promising photovoltaic materials. The top efficiency of perovskite based solar cells is beyond 22%^1^. It benefits from rich free carriers and their long diffusion distance up to microns^2–4^. It has been proposed and proved that the exciton and free carriers dynamically co-exist within the perovskite films^5,6^. However, this model seems incomplete, since the electrons and holes are both free carriers within the working layer, while their recombination rate is low. A study presented a relative high barrier of 75 meV for their recombination^7^, indicating that the carriers are not moving or recombining freely inside the material. Theoretical analysis suggested that the ferroelectric domains exist inside perovskite crystal grains of a film, while the electron and hole are spatially confined and transported within different tunnels formed between domains^8^. Therefore, a more reasonable analysis should consider the internal domains as the reason for low combination rate and high efficiency. In fact, the subgrain domains had been widely investigated in inorganic perovskites^9–11^. Very recently, ferroelastic/ferroelectric subgrain domains were discussed in organolead perovskite^12,13^. However, the wide existence of subgrain structure in real working layers is hard to be proved because of the surface roughness^13^. The discussion on substantial influence of the subgrain structure then cannot continue.

It is already known in material science that the photoproduct and morphology are correlated. In low dimensional semiconductors, some intrinsic hidden photoproducts become visible. E.g., in CsPbX_3_ perovskite quantum dot system, the charged-exciton and bi-exciton were found^14^. The relationship between morphology and photoproduct system is ‘cause and effect‘. Though the change of morphology may not lead to the variation on photoproducts, the photoproduct variation comes from the change of morphology. Therefore, studying the photoproducts is a reasonable way to disclose the change of morphology.

In this report, we examined the photoproducts and their relations in differently proceeded perovskite films, with our recently developed density-resolved spectroscopic method^6^. This method directly monitors the density-dependent (the total density of exciton and pair of free carriers) behavior of photoproducts to identify them and their interconversion. In the freshly made, non-heat annealed CH_3_NH_3_PbI_3_ film, exciton and free carriers interconvert to each other and form dynamical co-existing relation. However, when the films are sufficiently heat annealed, an additional decay channel of emissive exciton-carrier collision (ECC) appears and becomes dominant at high excitation density. The analysis revealed that an additional level of internal morphology within the crystal grain is responsible for the emergence of ECC. Such subgrain morphology can be attributed to a ferroelastic domain structure discussed very recently^12,13,15^. Since the heat annealing is a general applied procedure in preparing perovskite working layer in high performance devices, and the appearance of ECC is robust in annealed films, we conclude that the ECC and corresponding subgrain domain widely exist. Both our new experimental method, the newly found photoproduct system, and the proved subgrain structure in real working layers are crucial for in depth understanding the fundamental mechanism for the high performance of the devices.

## Method and Results

**Figure 1:**
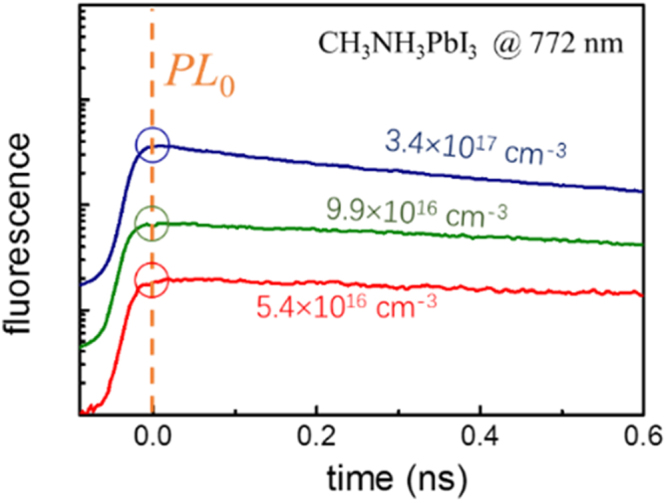
The typical fluorescent decay of CH_3_NH_3_PbI_3_ films after excitation. The films were pumped by a femtosecond laser at 517 nm with various pulse energy, while the peaks of fluorescence are ~772 nm. The *PL*_0_ is taken at the maxima for each fluorescent decay (circled). The corresponding excitation densities are labelled for each decay.

The density-resolved spectroscopic method is described as following. The CH_3_NH_3_PbI_3_ perovskite films were excited at 517 nm. The excitation densities, *n*, were calculated according to the injected photons per pulse and the illuminated volume of the thin films. Due to the fast and long range hot-carrier transportation, the photoproducts are supposed to be uniformly distributed in the volume^16^. The *n* is between 1×10^16^ and 5×10^18^ cm^−3^. As shown in Fig. 1, the spectra at temporal maxima of photoluminescence, *PL*_0_, is collected. A detailed description can be found in our former report^6^.

**Figure 2:**
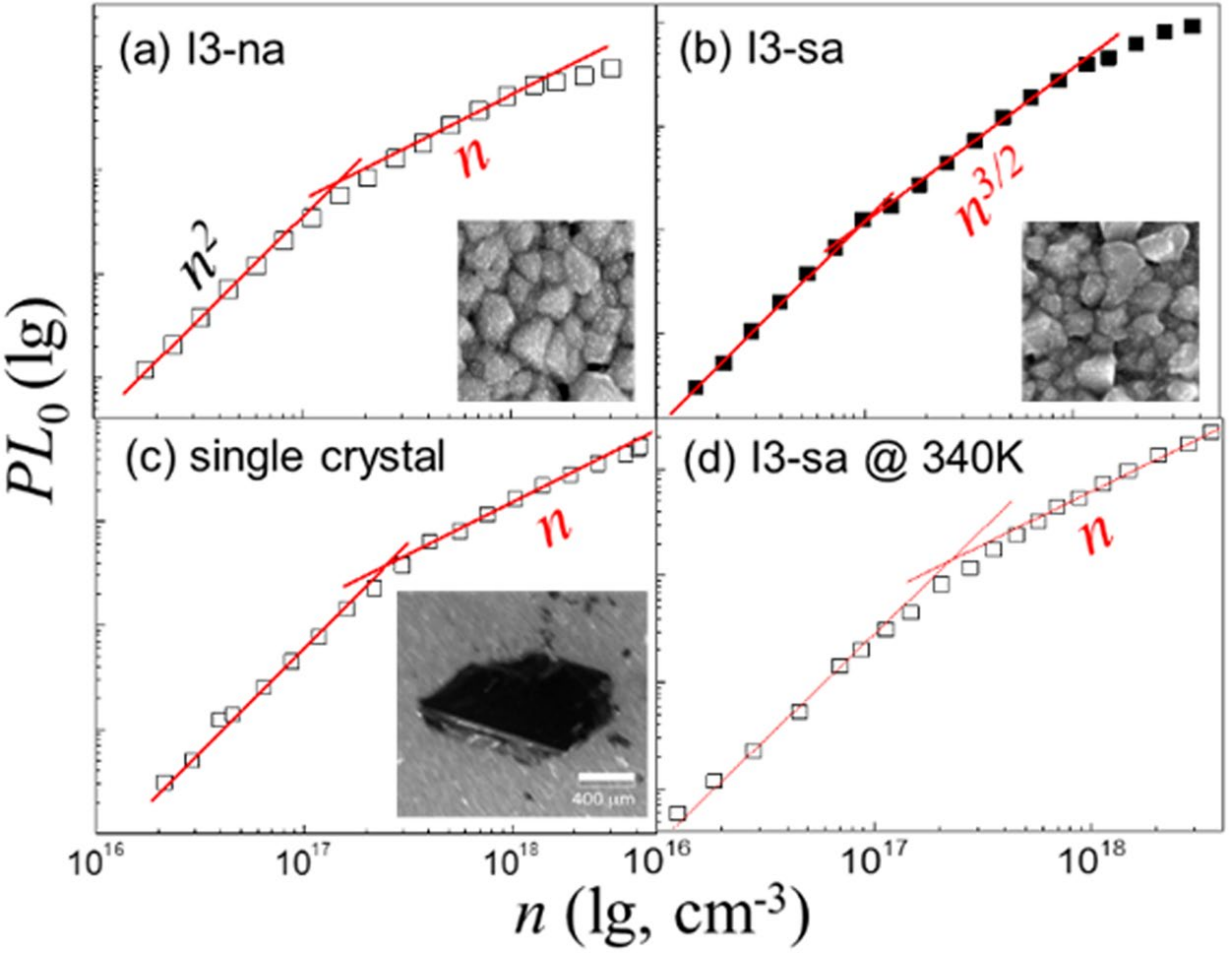
*PL*_0_ versus excitation density *n*. (**a**) and (**b**) are the I3 films without/with annealing, respectively. The film thickness is ~250 nm. The straight red lines are drawn with strict slop of 2, 3/2, and 1, for visualization. Corresponding SEM images are of 1×1 μm^2^. The *PL*_0_-*n* curve for single crystal are also drawn, with density estimated according to a film of ~250 nm thickness. (This estimation will not affect the power dependencies) The scale bar is 0.4 mm (**c**). The *PL*_0_ of I3-sa film at 340 K is recorded (**d**).

The samples are CH_3_NH_3_PbI_3_ (I3) perovskite films, which were either non-heat annealed (I3-na) or with sufficient annealing (I3-sa). A piece of single crystal was also tested. The amplitude of *PL*_0_ has power law dependency to *n*, as shown in Fig. 2. When *n* is lower than 10^17^ cm^−3^, the dependencies are quadratic for every sample. At higher density, the dependencies are clearly different. The I3-na, the single crystal, and I3-sa measured at 340 K (I3-sa@340 K) have a linear dependency (Fig. 2(a),(c) and (d) respectively), while the I3-sa measured at room temperature (I3-sa@RT) presents a 3/2 power index towards *n*. Similar power index of 3/2 can also be found in Cl doped I3 films (see Supplementary Information, SI). It should be noted that the power indexes are discrete values, which are integral multiples of 1/2. Therefore, the values of power index can be well distinguished without ambiguity.

## Discussion

A specified photoproduct can be identified by its density dependent behavior. The exciton and free carriers co-existing system has quadratic dependency at low excitation density. It become linear at high density. The critical density for power law conversion is decided by the exciton binding energy, *E*_B_^6^. Such dependency and index conversion can be well described by the Saha-Langmuir equation for a three-dimensional (3D) semiconductor^5^. The relative ratio of free carriers (electron or hole) in total excitation density, *x*, is written as,

1 $$\frac{{x}^{2}}{(1-x)}=\frac{1}{n}{(\frac{2{\rm{\pi }}\mu {k}_{B}T}{{h}^{2}})}^{3/2}{e}^{\frac{{E}_{B}}{{k}_{B}T}}$$

In this equation, the 1−*x* is the corresponding ratio of exciton. *μ* is the reduced effective mass of ~0.15 *m*_e_ (mass of electron^17^), *K*_B_, *T*, and *h* are the Boltzmann constant, the temperature, and the Planck’s constant, respectively. The fluorescence includes both monomolecular emission of exciton and bimolecular recombination of free carriers. It can be written as

2 $$I(n)\propto {A}_{1}(1-x)n+{A}_{2}{(xn)}^{2}$$

In this expression, *A*_1_ and *A*_2_ are the decay rate of monomolecular and bimolecular emission terms, respectively. *x* is from Eq. (1). By combination Eqs (1) and (2), the fluorescence quadratic increases at low excitation, which convert to linear dependency at high excitation density^6^. This means that the I3-na, single crystal, and I3-sa@340 K have similar photoproducts.

The I3-sa@RT presents a 3/2 power law dependency at high excitation density (Fig. 2(b)). The simplest and reasonable way is to attribute it to the presence of a new emissive decay channel, ECC. We add a corresponding term to Eq. (2),

3 $$I(n)\propto {A}_{1}(1-x)n+{A}_{2}{(xn)}^{2}+{A}_{3}(1-x)n\cdot xn$$

**Figure 3:**
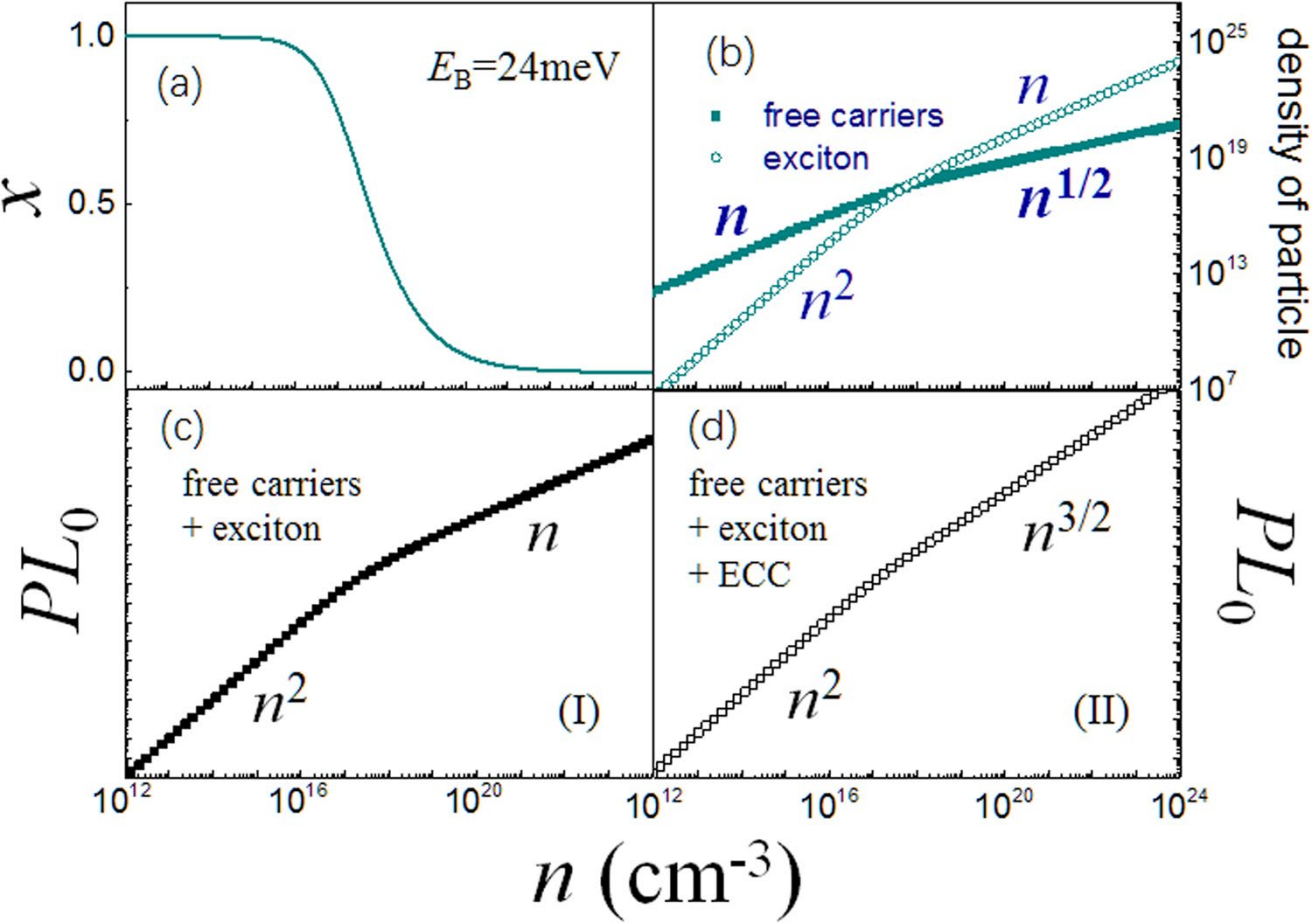
The simulation of density-dependent photoproducts inside perovskite films. (**a**) The relative ratio, *x*, of free carriers in total excitation density, according to Saha-Langmuir equation. (**b**) The simulated density of free carriers and excitons. (**c**) The simulated density-resolved *PL*_0_ when the free carriers and exciton dynamically co-exist inside perovskite, and (**d**) when ECC become dominant in the system.

*A*_3_ is the decay rate of the ECC. This term has a straightforward physical meaning that the ECC depends on the concentrations of both exciton and free carriers. This model generates the 3/2 power index at high excitation density. The simulation is shown in Fig. 3. Presented in Fig. 3(b), at high density ~10^17^–10^18^ cm^−3^, the carriers increase with 1/2 power to the density, while the exciton’s increment is linear, deduced from Eqs (1) and (2). Therefore the ECC generates the 3/2 power index at this density range, as shown in Fig. 3(d). Such kind of exciton-carrier interaction may possibly be expected as charged-exciton or trion^18^. However, a trion will not be stable in a 3D semiconductor, because of the weak binding energy (may less than 1 meV^19^).

The origination of ECC must be analyzed. The following well-known factors are ruled out: (1) The Auger process, which appears at high excitation density over 10^18^ cm^−3^ ^6,20,21^, with different power index to *n* ^22,23^. (2) The defects, which were estimated at the order of 10^15^ cm^−3^ ^4^, and below the quadratic section of bimolecular recombination^24^. (3) The external variation on film appearance, since the I3-na and I3-sa have no noticeable difference before and after heat annealing. (4) The unreacted PbI_2_ domains, which are usually found in non-heat annealed films^25^, but absent in the well-grown single crystals^26^. However, same co-existing relation was found for them, indicating the insignificance of the PbI_2_ domains. Therefore, by ruling out all other possibilities, a new level of intrinsic morphology must be proposed within the tetragonal crystalline structure of the crystal grain.

**Figure 4:**
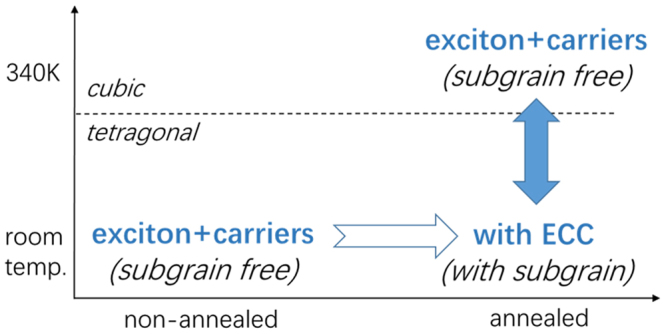
The photoproduct systems and their conversion for organolead perovskite films.

Such proposal is not created out of thin air. The subgrain domains had been observed in perovskite film as twinning structure in tetragonal phase. It disappears when the film was heated above the phase transition temperature to cubic phase, and re-appears after cooling down to room temperature^15^. The ECC has a similar dependence, disappearing at cubic phase (340 K, the phase transition temperature is ~330 K), and re-appearing when the crystal grains cooled down. In addition, to trigger the generation of ECC, heat annealing is required to proceed I3-na into I3-sa. This first time heat annealing brings the I3-na films into cubic phase and cools to tetragonal phase. Then the ECC will replace the co-existing thereafter. This is also the way to generate twinning structure in the inorganic perovskite films^10^. After this initial heat annealing, the subgrain structure appears and will not be erased in tetragonal phase. These similarities are the solid base to our analysis. The conversion of photoproducts and proposed subgrain structure are summarized in Fig. 4. With both experimental proofs and analysis, we concluded that our assignment is reliable.

It should be noticed that the universality of subgrain domains are hardly to be proved. Besides the surface roughness which can hinder the observation, the domains may have variable size following square root law^27,28^, variable orientation, and irregular shape other than twinning domain^13^. However, these differences should not affect the general opto-electronic properties of the films, since the high efficiency of organolead perovskite based solar cells can be easily repeated in countless labs worldwide, regardless of the differences on these morphology and processing methods. However, the current few studies, which clearly revealed subgrain morphology, require specialized sample films, such as flattop surfaces^12,13,15^. In contrast, the optical method can directly look into the films, regardless of the surface condition. The method revealed the ECC is a robust phenomenon in heat-annealed films. Therefore, wide existence of ECC and subgrain domains in films are then uncovered.

Frost *et al*. suggested that the domain walls are the pathways for carrier transportation, so that the electron and hole are transported in different tunnels^8,29^. It had been discussed that the subgrain domain boundaries may significantly affect the photophysical and electronic behaviors of organolead perovskite^30^. Such domain walls had been suggested with conductivity 3–4 orders higher than the bulk material^31^. The study by Colsmann *et* *al*. revealed charge extraction of photo-induced free carriers from the twinning ferroelectric structure^13^. Therefore, the long free carrier lifetime, slow recombination rate, and high conductivity in the perovskite films can be easily understood with the knowledge of such subgrain domains. We suggest that our discovery provides the morphological basis for understanding its high performance such as carrier transportation property.

In this report, we applied our newly developed density-resolved spectroscopic method to uncover two different exciton-free carrier relations. We found that when the films are newly made without sufficient heat annealing, the exciton and free carriers are dynamically balanced. This is very similar to the single crystal. However, for the films with sufficient heat annealing, the ECC appears, showing the exciton and free carriers do not co-exist peacefully. Our analysis and experimental proofs indicate that the appearance of ECC means the emergence of a subgrain domain inside the crystal grains, which could be assigned to the ferroelastic twinning structure. The results shows the ECC is a robust exciton-carrier relation. It is the major photophysical behavior at high density range, other than widely supposed co-existing relation. Then it also means the subgrain domains widely exist in crystal grains of the films. This is a fundamental discovery, possibly the key, for understanding the high device performance, such as long carrier lifetime and diffusion length. We also suggest that such method and discoveries can be applied for searching new perovskite materials for high performance photovoltaic applications.

## Electronic supplementary material

Supplementary Information
