# Supplementary material for: Interplay between Exciton and Free Carriers in Organolead Perovskite Films
Source: Sci Rep. 2017 Nov 7;7:14760. doi: 10.1038/s41598-017-15097-y (PMC5677142; doi:10.1038/s41598-017-15097-y)
Supplement: Supplementary file 1 — Supplementary Information [file 41598_2017_15097_MOESM1_ESM.pdf]

Supplementary information

Interplay between Exciton and Free Carriers in Organolead Perovskite Films

Wei Wang, [†,§] *Yu Li,* [†,§] *Xiangyuan Wang,* [‡] *Yang Liu, Yanping Lv,* [‡] *Shufeng Wang,** [,†] *Kai Wang,* [‡] *Yantao Shi,** [,‡] *Lixin Xiao,* [†] *Zhijian Chen,* [†] *and Qihuang Gong** [,†]

[†]State Key Laboratory for Artificial Microstructure and Mesoscopic Physics, Department of Physics, Peking University, Beijing 100871, China.

and Collaborative Innovation Center of Extreme Optics, Shanxi University, Taiyuan, Shanxi 030006, China.

[‡]State Key Laboratory of Fine Chemicals, School of Chemistry, Dalian University of Technology, Dalian, Liaoning 116024, China.

AUTHOR INFORMATION

Corresponding Author

* wangsf@pku.edu.cn; shiyantao@dlut.edu.cn; qhgong@pku.edu.cn

Sample preparation:

I3-na and I3-sa:
462 mg PbI2 (Sigma-Aldrich, 99.999%) was dissolved into 1 mL anhydrous dimethyl formamide (DMF, J&K, anhydrous, 99.8%) at 70ºC and then were spin-coated on a quartz substrate at 5000 rpm for 10 s. After heating at 70ºC for 30 min, they were cooled down to room temperature. Then the PbI2 films were dipped into a solution containing 10 mg CH_3NH_3I in 1 ml 2-propanol (IPA, J&K, anhydrous, 99.5%) for 2 min. Then the films were rinsed with 2-propanol, and dried by spinning at 3000rpm for 30s. The I3-na was left as it was, while the I3-sa was further annealed at 70ºC for 30 min.

For I3Cl-sa:
102.2 mg PbCl2 (Sigma Aldrich, 99.999%) and 174.9 mg CH_3NH_3I (with a molar ratio of 1:3) into 0.5ml anhydrous DMF and then heated at 70 C for 1h. $CH_3NH_3PbI_{3-x}Cl_x$ films were fabricated by spin-coating of the precursor solution at 2000 rpm for 30 s. The I3Cl-sa was annealed at 90ºC for 1 hr. and 100 ºC for 25min.

Crystallization of $CH_3NH_3PbI_3$:
2.5 g $PbAc_2$ and 10 ml hydroiodic acid (HI) 57 wt% in water were added into a tube. The tube was heated at 85 °C to dissolve the solution completely. Black precipitates came out when a solution, containing 2 ml HI and 0.597 g 40 wt% CH_3NH_2 in water (Merck), was added. Single crystals were grown along with the slow cooling of the solution from 85 °C down to 46 °C.

More discussion for photoproduct conversion.

For exciton and free carriers dynamical co-existing photoproduct system, System I

For perovskite system containing free carriers and exciton, such as freshly made I3-wa and single crystal, the dynamical co-existence of the two photoproducts follows Saha-Langmuir equation, shown in main text and Eq. S1 here,

$$\frac{x^2}{1-x} = \frac{1}{n}\left(\frac{2\pi\mu k_B T}{h^2}\right)^{3/2} e^{-\frac{E_B}{k_B T}} = \frac{1}{n}C(T, E_B) \qquad \text{(S1)}$$

In this equation, x is the ratio of free carriers in total excitation density, n. The corresponding 1-x is the ratio of exciton in total density, n. μ is the reduced effective mass of ~0.15 m_e (mass of electron [1]), K_B, T, and h are the Boltzmann constant, the temperature, and the Planck's constant, respectively. E_B in this equation is the exciton binding energy.

From Eq. (1), we can derive the x at low and high excitation limit. The Eq. (1) indicates that at the low limit where the free carriers are rich, $x \to 1$ and $x^2/(1-x) \to 1/(1-x) = C/n$. Then the (1-$x$) is proportional to n. On the other hand, at high excitation density where exciton takes the main role, $x \to 0$ and $x^2/(1-x) \to x^2 = C/n$. Then the x is proportional to $(1/n)^{1/2}$. This knowledge can also be found by theoretical calculation.[2] It should be motioned here that the C means the conversion density from the free carrier rich to the exciton rich situation.[3] The low and high excitation density limit means $n \ll C$ and $n \gg C$, respectively.

When the system containing photoproducts of exciton and free carriers, the fluorescence can be written as the Eq. 2 in main text, which is also copied here as S2

$$I(n) \propto A_1(1-x)n + A_2(xn)^2 \qquad \text{(S2)}$$

In this expression, A_1 and A_2 are the decay rate of mono-molecular and bi-molecular emission terms, respectively. x and n are taken from Eq.(S1). At low and high excitation density, the Eq.(S2) include two components representing the emissive decay of exciton and bimolecular recombination, respectively. At low and high excitation density, $x \to 1$ and 0, where the S2 will be S3 and S4, respectively.

$$I(n) \propto \frac{A_1}{C} \cdot n^2 + A_2 n^2 = \frac{1}{C}(A_1 + A_2 C)n^2 \qquad \text{(S3)}$$

$$I(n) \propto A_1 n + A_2 C n = (A_1 + A_2 C)n \qquad \text{(S3)}$$

The equation clearly shows quadratic dependency at low excitation density and linear dependency at high density. With this knowledge, an exciton and free carrier balanced system can be derived.

With the presence of exciton-carrier collision (ECC), System II

When ECC appears, one more term should be added to Eq. S2

$$I \propto A_1(1-x)n + A_2(xn)^2 + A_3(1-x)n \cdot xn \tag{S4}$$

As the former analysis, at high excitation density, the free carrier density, $xn \propto n^{1/2}$. On the other hand, the exciton linearly increases to the density, $(1-x)n \propto n$. Then the ECC term will present a 3/2 power law dependency to the density. At high excitation density, it becomes the major source of density dependent fluorescence. At low excitation density, since the third term is small comparing to the first two terms, the PL_0 still have the quadratic dependency to the density. A simulation can be seen in the following part.

Simulation of density dependent photoproduct behavior

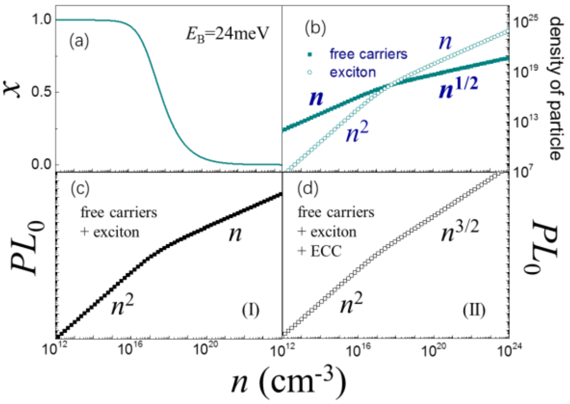

Figure S1. This figure is same to Figure 3 in main text, for the purpose of better reading.

The power index of the photoproducts and PL_0 towards n are summarized in Table S1.

Table S1. The density-dependent photoproducts and PL_0.

density	fc	exci	ECC	PL_0
$n < 10^{17}$cm^{-3}	~ $\boldsymbol{n}$	~ $\boldsymbol{n^2}$	~ n^3	~ n^2
$n > 10^{17}$cm^{-3}	~ $n^{1/2}$	~ n	~ $\boldsymbol{n^{3/2}}$	~ $n^{3/2}$

fc: free carriers. exci: exciton. The dependency of exciton and free carriers can also be found in Ref. [2]. The main photoproducts and decay channels at each density range are marked with bold font.

However it should be noticed that, from low density to high density, the transition from quadratic to 3/2 power law dependency in Fig. S1(d) may not be a smooth turning. As simulated below, we present three curves showing that A_3=A_2, A_3=10A_2, and A_3=0.1A_2. The results are shown in Figure S2. Such phenomena can be slightly observed in Fig. 2(b) and Fig. S3.

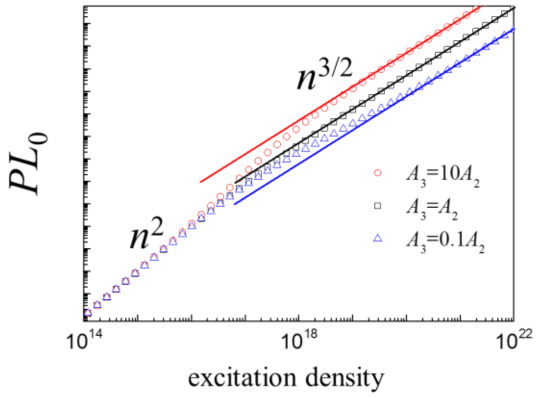

Figure S2. The simulation of Eq. S4, supposing A_1=0. The A_2 and A_3 are of different ratio.

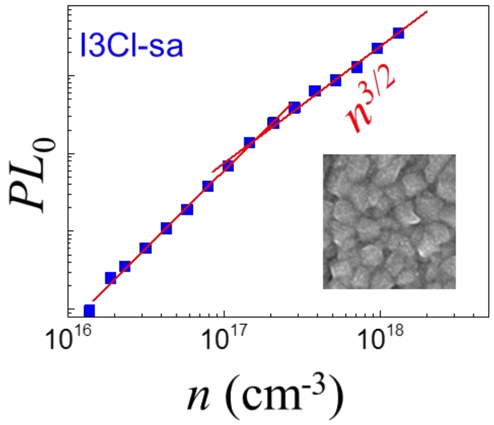

Figure S3. The PL_0 vs n for chlorine doped perovskite films. The SEM image of 1×1 μm^2 are inserted in the figure.

References

1. Tanaka, K.; Takahashi, T.; Ban, T.; Kondo, T.; Uchida, K.; Miura, N. Comparative study on the excitons in lead-halide-based perovskite-type crystals CH3NH3PbBr3 CH3NH3PbI3. *Solid State Communications* **2003,** *127* (9-10), 619-623.
2. Phillips, R. T.; Nixon, G. C.; Fujita, T.; Simmons, M. Y.; Ritchie, D. A. EXCITONIC TRIONS IN UNDOPED GaAs QUANTUM WELLS. *Solid State Communications* **1996,** *98* (4), 287.
3. Wang, W.; Li, Y.; Wang, X.; Lv, Y.; Wang, S.; Wang, K.; Shi, Y.; Xiao, L.; Chen, Z.; Gong, Q. Density-dependent dynamical coexistence of excitons and free carriers in the organolead perovskite CH3NH3PbI3. *Physical Review B* **2016,** *94* (14).
